# Design, validation and piloting of clinical vignettes to analyze critical care clinical decision processes during the COVID-19 pandemic in three different countries (Spain, Chile and United States)

**DOI:** 10.21203/rs.3.rs-3208463/v1

**Published:** 2023-08-10

**Authors:** Fabiola Jaramillo-Castell, Sergio Minué Lorenzo, Demetrio Carmona Derqui, Matthew Murphy, Carmen Fernández Aguilar, Kapil Nanwani, Manuel Quintana-Díaz, José Jesús Martín-Martín

**Affiliations:** Government of Chile Ministry of Health: Gobierno de Chile Ministerio de Salud; Escuela Andaluza de Salud Publica; University of Granada: Universidad de Granada; Brown University Public Health Program: Brown University School of Public Health; University of Granada: Universidad de Granada; La Paz University Hospital: Hospital Universitario La Paz; La Paz University Hospital: Hospital Universitario La Paz; University of Granada: Universidad de Granada

**Keywords:** Heuristics, Cognitive biases, Clinical decision making, Diagnostic error, Intensive care Unit, Critical Care

## Abstract

**Background:**

The use of heuristics in clinical decision-making processes increases in contexts of high uncertainty, such as those in Intensive Care Units (ICU. Given the impossibility of empirically studying their impact on real-world conditions, clinical vignettes were developed with the goal of identifying the use of heuristics in the care of critically ill patients during the COVID-19 pandemic in different clinical contexts.

**Methodology:**

Vignettes were designed by critical care physicians in Spain to assess the use of representativeness, availability, and status quo heuristics in the care of critically ill patients during the COVID-19 pandemic. The construct, internal and external validity of the vignettes designed in Spain, the United States and Chile were evaluated. A questionnaire was piloted with the vignettes being validated in the three aforementioned countries through a computer application built for this purpose.

**Results:**

16 study vignettes grouped into 5 models were created: each model included between 2 and 4 vignettes. The vignettes designed were closed-response vignettes with 2–3 possible alternatives. The vignettes, initially developed in Spain in Spanish, were translated to English and adapted to the Spanish used in Chile. The clinical content of the vignettes was not modified during the translation process.

**Conclusions:**

The vignettes allow for the study of the use of heuristics in critical care clinical decision making in the context of the COVID-19 pandemic. The piloting and validation process used can serve as a model for similar multinational studies exploring clinical decision making.

## Background

Clinical decisions are subject to wide variability depending on the degree of uncertainty about patient’s clinical condition as well the physician’s decision-making process. Knowledge about the use of heuristics (1) in the clinical decision-making process is very limited (2). Although a solid conceptual framework has been developed (3), empirical evidence on the relationship between the use of heuristics and diagnostic error is scarce (4).

Critical care is a setting where the possibility of making a diagnostic errors (DE) and/or therapeutic errors (TE) is high: in a retrospective analysis of 31 studies analyzing 5,863 autopsies in United States (US), 28% of autopsies identified a DE and 8% of the total were Class I (severe) errors, which suggests that about 45,000 deaths could occur annually due to DE in Intensive Care Units (ICU) in US (5). A recent systematic review published by AHRQ (Agency of Healthcare Research and Quality), estimates the existence of DE in 5.7% of all visits to emergency departments (ED) (6). However, studies on DE and their possible causes receive little attention from research funding agencies and scientific journals.

During the COVID-19 pandemic, healthcare providers faced great pressure in ED and ICU, having to make critical decisions quickly and with limited resources. It is important to know this uncertain environment favours the use of heuristics in clinical decision processes.

Clinical decision-making process is understood as a cognitive-behavioral process that involves the use of knowledge and skills acquired through a professional’s experience. These decisions may involve diagnostic, prognostic or treatment processes. Among the methods for investigating the clinical decision-making process, three are particularly noteworthy (7): clinical records audit, the study of claims and standardized patient care (Table 1). Clinical vignettes have been used for decades in), in a wide range of disciplines (8, 9), including marketing (10), economics (11), psychology (12), sociology (13) and education (14). There are many studies which use vignettes in medicine, whether in terms of quality measure (15), processes and lifelong learning, general practice (16). They are also increasingly used to analyze the variability of clinical decision-making including the study of cognitive biases (17).

Clinical vignettes “simulate” a real clinical situation in a hypothetical patient through a narrative description, from which study participants are asked to respond to one or more questions. Study design using vignettes can be *between-subject*, where participants are randomly assigned to different groups of vignettes, *within-subject*, where all participants are assigned the same vignettes, or mixed. There is also the possibility of using factorial surveys (18). Vignettes can be administered in person, by telephone or electronically (19). Several studies have demonstrated a high external validity of vignette studies in clinical decision processes when compared with a gold standard of standardized patients (7). However, it should be noted that the ecological (external) validity of this type of study remains inconclusive (20).

Given the importance of advancing our understanding of critical care decision-making processes, and given the practical and ethical impossibility of carrying out a study in real-world conditions during the COVID-19 pandemic, a study using clinical vignettes is a methodologically rigorous and scientifically valid approach to addressing this key knowledge gap.

## Methodology

Recommendations for the design of vignettes (7) (15) were followed for this study:

### 1.- Selection of the study heuristics

Heuristics previously studied in critical care and potentially relevant during the COVID-19 pandemic were selected.

### 2.- Search and Design of vignettes

A literature search was conducted with the aim of identifying validated vignettes for use in critical care decision-making. This search was complemented by the design of vignettes for the study.

### 3.- Assessment of the validity of the selected vignettes:

It was assessed the construct, internal and external validity. Construct validity (21) is the degree to which a variable measures the intended theoretical construct. In this study, that means whether the developed vignettes represent cases that would be encountered in the real world. Internal validity is the degree to which changes in the dependent variable can be accurately attributed to changes in the independent variable. External or criterion validity means that this study, using vignettes, produces results which can be generalised to real-world situations.

### 4.- Piloting-(pre-test evaluation). of selected and validated vignettes:

The vignettes were piloted with professionals similar to those of the target study population to verify that the instructions are correctly understood and the vignettes are adequately interpreted.

### 5.- Administration:

In this study, which focuses on the decision-making process, it is especially relevant to emphasize that the aim is not to evaluate knowledge or patient outcomes, but rather the way in which study participants would act in a similar situation. Ensuring responses are collected anonymously was emphasized.

Using the criteria found in the literature for the construction of vignettes (18, 22), a checklist of requirements which should be met was drawn up to evaluate the design of the vignettes in this study (Table 2).

## Results

### 1.- Selection of the study countries

The study was conducted in Chile, Spain, and the United States, which were chosen as members of the Organization for Economic Cooperation and Development (OECD). They cover the spectrum of alternative health system modalities, from a market-centred model (United States) to a “Beveridge-type national health system” model (Spain) (23). Chile represents intermediate characteristics between the two. The inclusion of these countries with diverse health system models allows for a comprehensive assessment of clinician decision-making.

### 2.- Selection of the study heuristics

Three heuristics were selected based on their use in previous studies in critical care decision-making by means of vignettes: availability, representativeness, and status quo. *The availability heuristic* (24) is a mental shortcut by which one judges the likelihood of an event in terms of its ease of recollection and retrieval from memory. *The representativeness heuristic* (25) estimates the probability of an event, e.g., a diagnosis, by comparing it to a prior stereotype which comes to mind. Finally, the *status quo heuristic* is defined as a tendency, not to modify a diagnosis or treatment, for fear of causing harm, even though the new diagnosis or course of action is more likely to be successful than the existing option (26).

### 3- Selection of the study vignettes

Two studies applicable to a pandemic scenario were obtained (27, 28).

Development of new vignettes: Two ICU physicians (KN and MQ) designed vignettes following the revised methodological criteria, based on their own professional experience. In order to adapt the vignettes to the possible use of the selected heuristics, they worked together with experts in behavioural economics (JJM, CF, MC, SM). In the construction of the set of vignettes, the following characteristics were taken into consideration:

- Hour of the day when the clinical decision occurred
- Age of the patient
- Wave of the pandemic: Different decisions between the first and the last wave may have been the result to the knowledge acquired during the interim and to the evolution of the pandemic itself (lethality of the disease, vaccination rates etc.)

As a result of the vignette design process, a total of 16 vignettes were created and grouped into 5 different models according to the following criteria (Table 3):

- Model 1: the two vignettes employed differ exclusively in the time at which the clinical decision occurs (case A, at 10:00AM and case G at 3:00 AM) as the response is thought to potentially change. The proposed hypothesis would be that at 3:00 AM, a heuristic-mediated response might be used more frequently.
- Model 2 (cases B,F,I,M) and 3 (cases C,J,L,P) includes 4 vignettes which differ in age of the patient and pandemic wave.
- Model 4 (including 4 vignettes D,H,K,N) and 5 (including 2 vignettes) which differ in pandemic wave.

The vignettes are not in correlative order in each model. This prevents the presentation of all the vignettes in the same model together. The correspondence of each vignette with each of the heuristics used in its response is shown in Table 4.

### 4. Validation of the vignettes

The vignettes underwent validation by experts in critical care, behavioural economics, and qualitative methodology. Experts in behavioural economics and qualitative methodologies remained consistent throughout the study. It was involved critical care experts in Spain, Chile, and the United States, to ensure the vignettes aligned with the clinical context, resulting in three sets of vignettes, one for each country, which represented situations which could exist in the real world according to critical care experts, supporting their compliance with *construct validity*. Likewise, the experts from the three countries affirmed that they would respond to the cases presented in the vignettes in the same way as they would in real situations, a criterion of compliance with *external validity*. Lastly, in each of the vignettes, at least one of the alternatives posed the possible use of a heuristic, according to behavioural economics experts, a criterion of internal validity (Table 4).

### 5.- Piloting.

The administration of questionnaires using vignettes on critical care was piloted in Spain at an Intensive Care Conference in March 2022. A total of 41 physicians responded, which included trainees and specialists in Intensive Care Medicine. As a result, the established response times and the content of the vignettes were modified. The mean response time for the complete questionnaire was 12 minutes (range 7–18).

The vignettes were subsequently piloted in Chile (Puerto Montt Hospital) and the United States (Rhode Island Hospital), and adaptations had to be made in the wording of the vignettes for each of these countries. In the case of the United States, an English translation was done by a professional translator and later adapted by a physician specialist in Internal Medicine and whose native language is also English (MM). Language and technical term adaptations were also made in Chile after the pilot test.

### 6.- Development of a computer application for completion

One of the members of the research team (DC), expert in programming, developed a beta application software for piloting. It was piloted in Spain with students of the Public Health and Health Management Master’s programmes (20 students) and Health Economics Master’s programme (25 students), both at the University of Granada / Andalusian School of Public Health. In the United States, it was performed by intensive care specialists who currently worked or previously had worked at Rhode Island Hospital/the Lifespan Healthcare system, a Brown University affiliate. In all three scenarios, adequate functioning of the application was verified, as well as the feasibility of completing the questionnaire, even with a high number of simultaneous users.

### 7.- Request for authorization from ethics committees in Spain, Chile and the United States.

The process of authorization in this matter was initiated through the ethics committees at Hospital La Paz in Madrid and the Provincial Ethics Committee of Granada in Spain. Subsequently (May 2021), authorization was requested and obtained from the Health Care Ethics Committee at the Reloncaví Health Service (Ministry of Health,Chile), which gave permission for the study to be performed in the whole of the Chilean territory. Finally, ethical approval was requested from the Institutional Review Board at Rhode Island Hospital/the Lifespan Healthcare System in the United States, a Brown University affiliate.

### 8.- Administration of the questionnaires

Once the questionnaires had been prepared, validated, piloted, adapted electronically and authorised by the respective ethics committees, an intra-subject administration (within-subject) was chosen, so that all study participants will received the same vignettes in a random order. The questionnaires were anonymous with any possible identification of the respondent restricted during the data collection processes. However, sociodemographic parameters were included for the comparative data analysis (Table 5).

## Discussion

During the COVID-19 pandemic, critical care professionals had to make decisions under time pressure, with an even greater level of uncertainty, as the disease was previously unknown and the resources necessary for its care (mechanical ventilators, intensive care beds) were unavailable to all patients, and on many cases they had to ration or prioritise said resources. In this context, it is very likely that heuristics were used in this process leading to a possible increase in the existence of biases (29). Three heuristics could have played an important role during the course of critical care response during the course of the pandemic: the possible bias of choosing to maintain an existing situation for fear of causing harm (status quo/omission bias), the bias of diagnosing all cases with certain symptomatology as COVID-19 (representativeness) or that the most striking cases recur much more frequently than their real prevalence rate (availability).

The three selected healthcare systems (Chile, Spain, United States) are only representative of themselves, but the study in three such different countries could provide preliminary information on whether the use of heuristics is widespread in medical practice. Analyzing the decision-making process of critical care physicians, and whether this process led to preferably analytical or intuitive decisions, is difficult to carry out in real conditions. In this case, clinical vignettes are indicated to fulfil this objective.

In order to optimize the construct, internal and external validity of the vignettes, they were subjected to assessment by experts representing both clinical practice and behavioural economics. In both groups of experts, a substantial lack of knowledge was observed with respect to the area of study of the other group: while the lack of familiarity among economists of the care of critically ill patients is perhaps more understandable, the lack of knowledge of the term heuristic and its meaning among the clinicians in the pilot groups in the three countries was striking. This also meant there was a need for an introduction to key study concepts prior to its distribution among clinicians. Sufficiently clear prior instructions about the study is central to successful and accurate completion.

Although it was necessary to adapt the vignettes prepared in Spain to the medical language commonly used in Chile and the United States, there was no questioning of the clinical cases proposed in the vignettes in these two countries.

In the process of constructing the vignettes, we opted for the closed-response question modality with two or three alternatives, as it was considered to facilitate the identification of heuristics better than an open-response questionnaire. We also opted for within-subject administration, in which all participants in the study received the same vignettes instead of establishing groups of vignettes randomly distributed to different groups, to allow for a broader exploration of the decision process.

The presentation of the vignettes in different conditions (hour, age of patients, pandemic wave) increases the diversity of decision scenarios, broadening their complexity and approximating the variety of real-world situations, as a tool to increase their external validity.

By systematically following the recommendations from the existing literature regarding vignette development and the absence of substantial proposed modifications during the validation and piloting process of the vignettes, these vignettes could be considered a useful instrument for the study of clinical decision making in critical care during the COVID-19 pandemic in other contexts after a process of adaptation and validation.

The complexity of the process of requesting review by the ethics committees in each country, each with different requirements and conditions, should be emphasised. It would be advisable to have internationally standardised procedures for studies with these characteristics.

Studies using clinical vignettes have *limitations*, including those related to their construct and external validity. As they are hypothetical scenarios, they are exempt from the conditioning factors and determinants of real clinical practice, such as time limitations and the pressure which exists at time of decision. This study attempted to minimize this potential limitation. It should be noted that this study, similar to other studies of this type, is subject to the same limitation of non-respondent bias (30).

Vignette studies are also subject to the Hawthorne effect (31). Another possible limitation is the sentinel effect (32): behaviour can be modified by the fact that it is being evaluated. An attempt was made to reduce this possible limitation by guaranteeing complete anonymity of the responses. It was also reiterated that the study was in no way evaluative of clinical knowledge or patient outcomes but was simply for the purposes of gaining insight into the clinical decision-making process.

The evidence available on the validity of vignette studies comes mainly from primary care settings (33), where results using this methodology correlate positively with real-world studies, thus constituting a proxy for evaluating practice. However, studies on conditions such as those seen in critical care, remain scarce (34). Despite the existing limitations of this type of methodology, it is still one of the few available approaches to research clinical decision-making in critical care.

## Conclusions

16 vignettes were developed of different clinical scenarios for the study of decision-making in critical care patients during the COVID-19 pandemic. The scenarios varied according to the time of day clinical care was given, the age of the patients evaluated and the pandemic wave in which the care was provided.

The vignettes were developed by critical care specialists based on existing literature and were validated by ICU specialists in Chile, Spain, United States. Both the vignettes and the computer application for their completion via the Internet were piloted in the three countries, with prior authorization from respective ethics committees.

Despite the limitations existing in this type of study, the set of designed vignettes allows for an approach to the study of clinical decision-making in critical care during the pandemic to evaluate the possible use of heuristics.

## Figures and Tables

**Table 1:** Methods of research approaches on clinical decision procedures.

Method	Characteristic	Strengths	Weaknesses
Medical Record Abstraction	Information available from clinical records/clinical history	Readily available information Records of actual interactions Low workload for clinicians	Recording bias (not all recorded information is relevant or reliable). Availability bias (not all physicians may authorise its use) Costly data extraction (in time and labour) Poorly systematizable Smaller samples (less generalisable results)
Claim Data Analysis	Information available from patient claims/statement records	Readily available information Records of actual interactions Reduced workload for clinicians Samples can be larger (results more easily generalised))	Recording bias (not all recorded information is relevant or reliable) Incomplete and often inaccurate information Costly data extraction (in time and labour) Difficulties to assign decisions
Standardised Patients	Medical behaviour in the presence of standardised patients (usually a trained actor)	Records of simulated interactions based on real cases Possible capture of interactions which cannot be recorded in the two previous methods.	Requires information collection by clinicians (increases workload) No records of actual interactions High cost (training, interaction performance) Logistic complexity Small sample sizes (representativeness difficulties) Not possible to simulate all interactions Participant bias
Clinical Vignettes	Medical conduct	Simulated interaction registration based on real cases Lower cost than standardised patients The information is the information necessary for decision making Easier to exploit the information collected More generalisability when large sample size	Requires information collection by clinicians (increases the workload). Small sample sizes (representativeness difficulties) Participant bias Replication to real-world conditions difficulties Social desirability bias: how they should respond rather than how they would act in reality Non-respondent bias Cost of validation and piloting

Source: Modified from Converse, 2015.

**Table 2:** Check list for Vignette Design

Scope	Recommendations	Results
Construction of the Vignette	Derived from literature and/or clinical experience.	An initial bibliographic search was carried out The authors of the new vignettes were intensive care specialists with more than 10 years of clinical experience.
	Be clear, well-written and carefully edited	Reviewed by experts in qualitative methodology, intensive care and behavioural economics.
	Be as concise as possible (usually between 50 and 500 words).	Mean 147. Median: 140. Range 104–180
	Follow a narrative progression similar to a story.	Present in all vignettes.
	Follow a similar structure and style for all the vignettes in the study.	Identical in all vignettes.
	Use present tense (past tense only for story and background information)	Present in all vignettes.
	Cases should seem like real people, not a personification of a list of symptoms or behaviours	Present in all vignettes.
	Use layouts which include both necessary and unnecessary attention	Present in each group of cases.
	Balance gender and age throughout the vignettes	2 females, 2 males, in 12 no sex was identified
	Be as neutral as possible with respect to cultural and socioeconomic factors	Confirmed by the three validation processes in the three countries.
Administration of Questionnaire	Allow for open-ended responses	Closed responses (2–3) were chosen as more appropriate for exploring the use of heuristics.
	Present realistic time constraints	The decision time limit was equal to or less than 10 seconds to approximate its response in real conditions.
	Suggest cases with varied and realistic levels of complexity	Presentation of heterogeneous cases in terms of content and complexity.
	Provide real time information on demand from physicians	Not applicable as carried out in three different state contexts, which would have required a great availability of economic resources, thus not permitting an adequate cost/rigor balance.

Modified from Peabody (2000) and Evans (2015)

**Table 3:** Models and Vignette modalities

Model	Case	Variations			
		Age	Wave	Hour	Reversal Test
1	A	44	1	10 AM	No
	G	44	1	03 AM	No
2	B	74	1	-	No
	F	54	Last	-	No
	I	54	1	-	No
	M	74	Last	-	No
3	C	81	Last	-	No
	J	45	1	-	No
	L	45	Last	-	No
	P	81	1	-	No
4	D	60	1	-	Yes
	H	60	Last	-	Yes
	K	60	Last	-	Yes
	N	60	1	-	Yes
5	E	34	Last	-	No
	O	34	1		No

**Table 4:** Distribution of the use of heuristics in vignettes according to type of response

Case	Heuristic	Model	Wave	Response		
				A	B	C
A	Status quo/Omission	Model 1	1	-	H	
B	Status quo /Omission	Model 2	1	-	H	H
C	Representativeness	Model 3	6	-	H	
D	Status quo/Omission	Model 4	1	-	H	
E	Availability	Model 5	6	H	-	-
F	Status quo / Omission	Model 2	6	-	H	H
G	Status quo/ Omission	Model 1	1	-	H	
H	Status quo/ Omission	Model 4	6	-	H	
I	Status quo / Omission	Model 2	1	-	H	H
J	Representativeness	Model 3	1	H	-	
K	Status quo/Omission	Model 4	6	H	-	
L	Representativeness	Model 3	6	H	-	
M	Status quo /Omission	Model 2	6	-	H	H
N	Status quo/Omission	Model 4	1	H	-	
O	Availability	Model 5	1	H	-	-
P	Representativeness	Model 3	1	-	H	

NOTES:

1-Model 2 (cases B, F I, M) explores the use of heuristics with three options instead of a dichotomous one like the rest.

-2.-In model 4 Reversal Test will be produced if:

--the respondent chooses option A in cases D or H (they choose to withdraw anticoagulation and suspend CT) with options A in cases K or N (they choose to maintain anticoagulation).

--the respondent chooses option A in cases D or H (choose to withdraw anticoagulation and discontinue CT) and option B (indicate CT) in cases K or N.

-That is, only in the choice of options B in cases D and H and with options A or B (any option in short) in cases K and N, would Reversal Test not occur.

**Table 5:** Sociodemographic data included in the questionnaire

Age
Sex
Resident/Specialist
Speciality
Year finished Specialisation
Year started in current unit
What type of contract do you have? (permanent/ temporary)
Have you been infected with SARS-Co V 2?
Were you aware of the term Heuristic before this study?
Do you have religious beliefs?
Do you usually work in the ICU?
Name of health centre
City
Country

## Data Availability

Not applicable.
